# Can Bats Serve as Reservoirs for Arboviruses?

**DOI:** 10.3390/v11030215

**Published:** 2019-03-03

**Authors:** Anna C. Fagre, Rebekah C. Kading

**Affiliations:** Department of Microbiology, Immunology, and Pathology, Colorado State University, Fort Collins, CO 80523, USA; anna.fagre@colostate.edu

**Keywords:** arboviruses, bats, reservoir, wildlife, zoonoses

## Abstract

Bats are known to harbor and transmit many emerging and re-emerging viruses, many of which are extremely pathogenic in humans but do not cause overt pathology in their bat reservoir hosts: henipaviruses (Nipah and Hendra), filoviruses (Ebola and Marburg), and coronaviruses (SARS-CoV and MERS-CoV). Direct transmission cycles are often implicated in these outbreaks, with virus shed in bat feces, urine, and saliva. An additional mode of virus transmission between bats and humans requiring further exploration is the spread of disease via arthropod vectors. Despite the shared ecological niches that bats fill with many hematophagous arthropods (e.g., mosquitoes, ticks, biting midges, etc.) known to play a role in the transmission of medically important arboviruses, knowledge surrounding the potential for bats to act as reservoirs for arboviruses is limited. To this end, a comprehensive literature review was undertaken examining the current understanding and potential for bats to act as reservoirs for viruses transmitted by blood-feeding arthropods. Serosurveillance and viral isolation from either free-ranging or captive bats are described in relation to four arboviral groups (*Bunyavirales*, *Flaviviridae*, *Reoviridae*, *Togaviridae*). Further, ecological associations between bats and hematophagous viral vectors are characterized (e.g., bat bloodmeals in mosquitoes, ingestion of mosquitoes by bats, etc). Lastly, knowledge gaps related to hematophagous ectoparasites (bat bugs and bed bugs (*Cimicidae*) and bat flies (*Nycteribiidae* and *Streblidae*)), in addition to future directions for characterization of bat-vector-virus relationships are described.

## 1. Introduction

Bats and the viruses they harbor have been of interest to the scientific community due to the unique association with some high consequence human pathogens in the absence of overt pathology. Virologic and serologic reports in the literature demonstrate the exposure of bats worldwide to arboviruses (arthropod-borne viruses) of medical and veterinary importance [1]. However, the epidemiological significance of these observations is unclear as to whether or not bats are contributing to the circulation of arboviruses. 

Historically, a zoonotic virus reservoir has been considered a vertebrate species which develops a persistent infection in the absence of pathology or loss of function, while maintaining the ability to shed the virus (e.g., urine, feces, saliva) [2,3,4]. Haydon et al. extended this definition of a reservoir to include epidemiologically-connected populations or environments in which the pathogen can be permanently maintained and from which infection is transmitted to the defined target population. The significance of the relative pathogenicity of the infectious agent to the purported reservoir host has been debated [5]. In the case of bats as a reservoir species, rigorous field and experimental evidence now exist to solidify the role of the Egyptian rousette bat (*Rousettus aegyptiacus*) as the reservoir for Marburg virus [6,7,8]. Considering arboviruses, additional criteria must be met in order to consider a particular vertebrate species a reservoir. Reviewed by Kuno et al., these criteria include the periodic isolation of the infectious agent from the vertebrate species in the absence of seasonal vector activity, and the coincidence of transmission with vector activity [9]. Further, the vertebrate reservoir must also develop viremia sufficient to allow the hematophagous arthropod to acquire an infectious bloodmeal [10] in order for vector-borne transmission to occur. Bats have long been suspected as reservoirs for arboviruses [11], but experimental data that would support a role of bats as reservoir hosts for certain arboviruses remain difficult to collect. Here we synthesize what information is currently known regarding the exposure history and permissiveness of bats to arbovirus infections, and identify knowledge gaps regarding their designation as arbovirus reservoirs.

## 2. Members of the Order *Bunyavirales*

The order *Bunyavirales* is divided into eight families, four of which pose threats to public health and veterinary medicine–families *Nairoviridae*, *Peribunyaviridae*, *Phenuiviridae*, and *Hantaviridae* [12]. While bats have been demonstrated to host hantaviruses, these viruses do not rely on an arthropod in their transmission cycle and thus will not be discussed [13]. Viruses in order *Bunyavirales* that have been experimentally examined in bats or described in field studies are descried in Table 1.

### 2.1. Family Nairoviridae, Genus Orthonairovirus

Members of the genus *Orthonairovirus* of medical and veterinary significance include Crimean Congo hemorrhagic fever virus (CCHFV) and Nairobi sheep disease virus (NSDV) [12]. CCHFV is transmitted by ticks in genera *Rhipicephalus* and *Hyalomma* [14]. While neither live virus nor nucleic acid of CCHFV has been detected from bats, serologic evidence suggests past infection of populations of bats across a diverse geographic range [15,16,17]. Further, bats are often parasitized by both soft and hard ticks, which occupy a diverse range of ecological niches in endemic countries [18,19,20]. A 2016 seroprevalance study by Müller and colleagues examining 16 African bat species (*n* = 1,135) found that the prevalence of antibodies against CCHFV was much higher in cave-dwelling bats (3.6%–42.9%, depending on species) than foliage-living bats (0.6%–7.1%) [15]. They also screened 1,067 serum samples by RT-PCR, but all were negative for CCHFV nucleic acid [15]. Experimental studies to assess the ability of bats to support replication of CCHFV have not been published.

### 2.2. Family Peribunyaviridae, Genus Orthobunyavirus

Members of the genus *Orthobunyavirus* include many viruses of importance to human and veterinary medicine, including Bunyamwera virus, California encephalitis virus, Jamestown Canyon virus, Kaeng Khoi virus, and La Crosse encephalitis virus [12], but limited evidence exists regarding the exposure or potential involvement of bats in the circulation of viruses in this family.

Kaeng Khoi virus (KKV) has been isolated from cimicid bugs (Order: Hemiptera, Family: Cimicidae) (*Stricticimex parvus* and *Cimex insuetus*) and from suckling wrinkle-lipped bats (*Tadarida plicata* ) in caves in Thailand, but was not isolated from soft ticks tested in the same area (*Ornithodorus hermsi*) [21]. Additionally, KKV has been implicated in the case of several mine workers who reported illness and were discovered to have seroconverted [22], demonstrating spillover of this virus to humans in association with the cave environment, and suggesting that cimicids may play a role in vectoring virus between bat and human hosts. To date, no experimental data have been generated to address this hypothesis.

Spence and colleagues attempted to experimentally infect Jamaican fruit bats (*Artibeus jamaicensis*) via intramuscular injection with Nepuyo virus (Group C serogroup), yet no infectious virus was subsequently recovered from the bats [23]. This is interesting considering two strains of Nepuyo virus were isolated from Jamaican fruit bats (*Artibeus jamaicensis*) and great fruit-eating bats (*Artibeus literatus*) in Honduras, and protective sera were found in Jamaican fruit bats in Trinidad. [24,25]. Bats of undetermined species were involved in a large serosurvey in Brazil that examined antibodies in wildlife against the Gamboa serogroup orthobunyaviruses, though none were found to be positive [26]. Seven and twelve species of Trinidadian bats were examined for antibodies by HI against Caraparu (Group C serogroup) and Maguari (Bunyamwera serogroup) viruses, respectively, and were all found to be negative [25].

### 2.3. Family Phenuiviridae, Genus Phlebovirus

Viruses in the genus *Phlebovirus* (family *Phenuiviridae*) of importance to human and animal health include Rift Valley fever virus (RVFV) and severe fever with thrombocytopenia syndrome virus (SFTSV) [12]. Bats of the species *Miniopterus schreibersii* (*n* = 1) and *Eptesicus capensis* (*n* = 2) were experimentally infected with RVFV and the *M. schreibersii* bat’s urine and liver tested positive for antigen [27]. A recent study by Balkema-Buschmann and colleagues experimentally infected Egyptian rousette bats (*Rousettus aegyptiacus*) with vaccine strain MP-12 and recovered infectious virus from spleen and liver of some animals [28]. Oelofsen & Van der Ryst (1999) examined 350 samples from 150 field-caught bats in Africa, yet none were positive for antigen by use of ELISA [27]. Kading et al (2018) detected neutralizing antibodies against RVFV in Egyptian rousette bats and little epauletted fruit bats (*Epomophorus labiatus*) in Uganda, a country that has recently experienced human cases of RVFV [29,30]. Whether or not bats serve as a reservoir of RVFV during interepidemic periods remains to be determined.

### 2.4. Unclassified Bunyaviruses

Bangui virus (BGIV) is an unclassified bunyavirus and was isolated from an unidentified bat in the Central African Republic (CAR) [31]. Mojuí dos Campos virus (MDCV) is another ungrouped bunyavirus isolated from an unidentified bat species [32,33].

## 3. Members of the Family *Flaviviridae*

The family *Flaviviridae* includes many high-consequence emerging arboviruses, including Zika virus (ZIKAV), yellow fever virus (YFV), and Dengue virus (DENV). Flaviviruses associated with bats that do not appear to utilize an arthropod vector (“no-known vector flaviviruses”) have been reviewed elsewhere [56]. Viruses in family *Flaviviridae* that have been experimentally examined in bats or described in field studies are descried in Table 2.

### 3.1. Dengue Virus

Interestingly, despite DENV isolations from *Artibeus* spp. bats in the wild, experimental infections of great fruit-eating bats (*A. intermedius*) with DENV-2 and Jamaican fruit bats with DENV serotypes 1 and 4 resulted in low levels of viremia, low rates of seroconversion, and lack of detection of viral RNA in the organs via RT-PCR, indicating that bats may not act as a suitable reservoir host [57,58,59]. Experimental infection of the Indian flying fox (*Pteropus giganteus*) resulted in no viremia or clinical signs, but intracerebral inoculation of little brown bats (*Myotis lucifugus*) resulted in irritability, paralysis, and death [60,61].

DENV nucleic acid and anti-DENV antibodies have been detected in Mexican bats on the Gulf and Pacific coast, and nucleic acid has been detected in the liver and/or sera of wild-caught bats in French Guiana [62,63]. Anti-DENV antibodies have been detected in multiple bat species in Uganda [29]. However, a survey in Central and Southern Mexico analyzing 240 individuals representing 19 bat species by RT-PCR resulted in no detection of viral nucleic acid [64]. A 2017 study by Vicente-Santos and colleagues examined 12 bat species from Costa Rica and found a cumulative seroprevalence of 21.2% (51/241) by PRNT and a prevalence of 8.8% (28/318) in organs tested by RT- PCR [65]. No infectious virus was isolated and viral loads were considered too low for the bats to function as amplifying hosts. Rather, Vicente-Santos and colleagues surmised a spillover event from humans to bats, with bats functioning as a dead-end host [65]. The serum of Jamaican fruit bats (*Artibeus jamaicensis*) and Great fruit-eating bats (*A. literatus*) from Grenada (*n* = 50) were also tested for antibodies against DENV 1, 2, 3, and 4, and none were seropositive [66]. While field evidence supports the exposure of bats to DENV in multiple geographic areas, experimental infections conducted to date are consistent in that bats are not likely to support DENV replication and circulation to levels high enough to infect blood-feeding mosquitoes. 

### 3.2. Japanese Encephalitis Virus

Multiple studies conducted experimental infections of insectivorous bats with Japanese encephalitis virus (JBEV) and found that bats were susceptible to infection with this virus. Three species of bats (big brown bats (*Eptesicus fuscus*), little brown bats (*Myotis lucifigus*) and Eastern pipistrelles (*Pipistrellus subflavus*)) were inoculated with JBEV in the laboratory and maintained infection while held under simulated hibernation conditions. Bats infected prior to hibernation were viremic upon arousing from hibernation, with circulating virus detectable as long as 112 days after the initial infection [67]. Big brown bats also demonstrated recurrent viremia in the absence of clinical signs in a subsequent study [68]. Importantly, researchers demonstrated a mosquito-bat-mosquito transmission cycle and postulated this may be an overwintering mechanism for JBEV since mosquitoes did successfully transmit JBEV to bats at low temperatures [67]. Eastern pipistrelles also became infected with JBEV after consumption of infected mosquitoes, demonstrating that bats could be infected orally as well as through a mosquito bite [67]. No demonstrable pathologic effects noted during infection of three bat species [big brown bats (*Eptesicus fuscus*), little brown bats (*Myotis lucifigus*) and Mexican free-tailed bats (*Tadarida brasiliensie mexicana*) with various strains of JBEV or St. Louis encephalitis virus (SLEV) [69]. No pathology nor viremia was appreciated when pipistrelles (*Pipistrellus abramus*) were infected with JBEV [70]. While experimental data demonstrated that some bat species can sustain JBEV infections and support mosquito-borne transmission of this virus, the epidemiological significance of these observations in the field remains unclear.

JBEV has been isolated from wild-caught bats in Taiwan (*Miniopterus fuliginosus* and *Hipposideros armiger terasensis* [32,71], China (*Rousettus leschenaultia* and *Murina aurata* [72,73], Japan (*Miniopterus schreibersi fuliginosus* and *Rhinolophus cornutus cornutus* [74]. Antibodies against JBEV have been detected in wild-caught bats in Indonesia (unspecified species) [75], China (*Rousettus leschenaultia, Cynopterus sphinx, Taphozous melanopogon, Miniopterus schreibersii, Pipistrellus abramus, Rhinolophus macrotis* and *Miniopterus fuliginosus* [76,77], Australia (*Pteropus scapulatus* and *Pteropus gouldi*) [78], Taiwan (unspecified species) [79], India (*Pteropus giganteus, Hipposideros pomona, Hipposideros speoris, Hipposideros bicolor, Hipposideros cineraceus, Megaderma lyra, Cynopterus sphynx*, and *Rhinolophus rouxi*) [80,81,82], and Japan (*Miniopterus schreibersi fuliginosus, Rhinolophus ferrum equinum Nippon, Vespertilio superans, Myotis macrodactylus, Rhinolophus cornutus cornutus, Pipistrellus abramus, Myotis mystacinus, Plecotus auritus sacrimontis*, and *Murina leucogaster hilgendorfi*) [83]. Multiple isolations of JBEV from locations where the virus is endemic, in addition to the fact that genetic characterization of isolates has supported their similarity to strains identified from human and mosquito isolates, support the role of bats in ongoing circulation of JBEV [84].

### 3.3. St. Louis Encephalitis Virus

Another medically-important flavivirus with both field-obtained information and *in vivo* experimental inoculation is SLEV. A 1983 study by Herbold and colleagues demonstrated that 9% of wild-caught *Eptesicus fuscus* and *Myotis lucifugus* (*n* = 390) in Ohio possessed neutralizing antibodies to SLEV [85]. Other serosurveillance efforts in North America and Grenada focused on detection of SLEV in free-ranging bat populations have resulted in largely negative findings [66,86]. Following experimental infection, viremia and transplacental transmission (albeit infrequent) was appreciated in Mexican free-tailed bats (*Tadarida brasiliensis*) [69,87]. The viremia in these bats reached 4 log units, likely too low a titer to facilitate transmission to a blood-feeding mosquito [10]. Upon inoculation, little brown bats (*Myotis lucifugus*) appear to be resistant or only slightly susceptible to SLEV [69]. Herbold and colleagues (1983) demonstrated that inoculation of *Eptesicus fuscus* with SLEV results in infection and virus was maintained throughout hibernation (70 days), with viremia developing four days following arousal (105 days post-infection) [85]. Low levels of viremia upon experimental inoculation in conjunction with low seroprevalence data indicate this virus likely does not utilize bats as a reservoir host in nature.

### 3.4. West Nile Virus

To date, biosurveillance testing of bats in Central America for WNV have turned up negative results. Grenadian *Artibeus jamaicensis* and *Artibeus literatus* (*n* = 50) bats were negative for WNV neutralizing antibodies by PRNT [66], 14 Trinidadian bat species (*n* = 384) were negative by ELISA for WNV antibodies [88], and 16 Mexican bat species (*n* = 146) tested for WNV RNA by RT-PCR were negative [89]. In North America, results have been negative or indicative of low levels of circulation in bat populations tested. Tissues from 312 field-collected bats representing seven species in Illinois tested by RT-PCR were all negative for WNV, and the same study reported one big brown bat (*Eptesicus fuscus*) with neutralizing antibodies (*n* = 97) [90]. A field survey taking place in New Jersey and New York reported one big brown bat and one northern long-eared bat (*Myotis septentrionalis*) with neutralizing antibodies to WNV (*n* = 83) [86]. In another field study, only two of 149 free-tailed bats (*Tadarida brasiliensis*) possessed neutralizing antibodies against WNV [91]. In Uganda, Kading et al. (2018) detected neutralizing antibodies to WNV in 2/8 African straw-colored flying foxes (*Eidolon helvum*), and 3/44 little epauletted fruit bats (*Epomophorus labiatus*) [29].

Simpson and O’Sullivan (1968) demonstrated experimental inoculation of African straw-colored flying foxes did not result in viremia though two of three bats developed neutralizing antibody. In the same study, two of three Egyptian rousette bats were infected but only trace viremia was detected and seroconversion was not appreciated [43]. Experimental inoculation of free-tailed bats (*Tadarida brasiliensis*) did not result in viremia, and infection of big brown bats resulted in low titers (10–180 PFU/mL) [91], not capable of supporting transmission to feeding mosquitoes [10].

### 3.5. Yellow Fever Virus

Attempts to experimentally infect vampire bats (*Desmodus rotundus*) and black mastiff bats (*Molossus rufus*) by mosquito bite (*Aedes aegypti*) were unsuccessful [11]. Experimental inoculation of multiple bat species (*Eumops perotis, Carollia perspicillata, Phyllostomus hastatus* and bats in the genus *Mollosus*) were similarly unsuccessful [92]. Still, Kading et al. detected a significant neutralizing antibody titer against YFV in one Egyptian rousette bat in Uganda in 2012, indicating bats are exposed to this virus in nature [29]. Uganda has experienced outbreaks of YFV in recent years [93].

### 3.6. Zika Virus

While multiple African bat species (*Eidolon helvum, Rousettus aegyptiacus,* and *Rousettus angolensis*) demonstrated viremia following inoculation with ZIKAV, *Mops condylurus* did not become viremic, although did contain low virus titers in the kidney [43,44]. Experimentally-infected little brown bats were susceptible to the ZIKAV by the intraperitoneal, intradermal, intracerebral and intrarectal routes of exposure, but not susceptible intranasally [94]. However, it is unclear how ZIKAV could circulate in bat populations. Kading et al. (2018) did not detect neutralizing antibodies to ZIKAV among 292 Ugandan bats screened. Flavivirus infections of bats with an emphasis on the potential role in Zika virus ecology has been reviewed elsewhere [95].

### 3.7. Other Members in Family Flaviviridae and Pan-Flavivirus Surveillance

Flavivirus serology has been historically challenging due to the cross-reactivity of viral epitopes to circulating antibodies [96]. Therefore, the results of serologic surveillance studies must be interpreted cautiously [29,97]. Further, multiple methods exist for antibody detection (e.g., HI, PRNT, ELISA), and the biological significance of neutralizing vs. non-neutralizing antibodies must be taken into account.

In 2010, the serum of 140 Mexican bats from three species (*Glossophaga soricina, Artibeus jamaicensis,* and *Artibeus literatus*) was assayed by PRNT using WNV, SLEV, and DENV 1–4, and 26 were positive for flavivirus-specific antibodies (19%). None of the titers exceeded 80, and all samples were also negative when tested for flavivirus nucleic acid by RT-PCR [97]. In a 2015 serosurvey, eight bats (2.6%) displayed non-specific hemagglutination-inhibition (HI) results indicating cross-reactivity or antibodies against an undetermined flavivirus [88]. Kading and colleagues performed a serosurveillance study in Ugandan bats and identified 13.6% (85/626) had non-specific flavivirus antibodies by plaque reduction neutralization assay (*Chaerephon pumilus*, *Hipposideros ruber*, *Mops condylurus*, *Nycteris macrotus*, *Eidolon helvum*, *Epomophorus minor*, and *Rousettus aegyptiacus*) [29]. Still, results generally supported the widespread exposure of bats in Uganda to flaviviruses [29].

In 2018, Sotomayor-Bonilla and colleagues reported that liver and spleen samples from 12 Mexican bat species tested negative using pan-flavivirus NS5 primers [98]. A recent study in Brazil suggested a lack of arboviral circulation in bat populations, as 103 individuals from 9 species were tested for molecular and serologic evidence of alphavirus and flavivirus infection and all were negative [99]. Results of experimental infection of Egyptian rousette bats with WNV and of Angolan free-tailed bats (*Mops condylurus*) with Ntaya virus resulted in very low levels of viremia, while infection of African straw-colored fruit bats with Ntaya virus resulted in neither pathology nor detectable viremia [43].

## 4. Members of the Family *Reoviridae*

Few studies have examined the presence of viruses in genus *Coltivirus* in bat populations, and to date, a single isolation has been made (Table 3) [127]. A 1984 study by Chastel and colleagues failed to detect antibodies to Eyach Virus (Reoviridae, Colorado Tick Fever group) in the serum of two field-caught bats [128]. To date, five orbiviruses have been isolated from wild-caught bats and serologic evidence exists for exposure of Australian and South American bats to orbiviruses (Table 3). While no evidence of human exposure exists for these bat-associated orbiviruses, Bukakata (BUKV) and Fomede (FOMV) appear to be strains of the Chobar Gorge species [129]. CGV was isolated from *Ornithodoros* species ticks in Nepal, and serum from nearby humans and ruminants possessed anti-CGV antibodies, indicating past exposure [130]. Further investigation is warranted to determine the true vector-host association of these viruses and their zoonotic potential. Viruses in family *Reoviridae* that have been experimentally examined in bats or described in field studies are descried in Table 3.

## 5. Members of the Genus *Alphavirus* (Family: *Togaviridae*)

Viruses in genus *Alphavirus* (family *Togaviridae*) that have been experimentally examined in bats or described in field studies are descried in Table 4.

### 5.1. Chikungunya

Enzootic circulation of CHIKV is understood to occur among non-human primates and forest-dwelling mosquitoes [142], but other vertebrates including rodents, bats, reptiles and amphibians have been shown to support CHIKV replication [143,144]. The range of peak viremia developed by big brown bats was relatively low, but within the range of infectivity to blood feeding mosquitoes [10,143]. When Indian flying foxes (*Pteropus giganteus*) and big brown bats were experimentally infected with CHIKV, bats developed viremia but no clinical signs of disease, indicating they could play a role in the natural transmission of this virus [60,143]. Experimental infection of African straw-colored flying foxes did not result in viremia or seroconversion to CHIKV, supporting a separate study which reported lack of viremia in experimentally infected Egyptian rousette bats and African straw-colored flying foxes [43,44]. In 2015, the serum of 42 wild-caught Grenadian bats (genus *Artibeus*) were subjected to PRNT and 15 (36%) were found to possess neutralizing antibody to CHIKV [66]. CHIKV has been circulating in Central and South America since 2013 [145]. Whether or not bats are contributing to the natural circulation of CHIKV in endemic areas or areas of introduction remains to be determined.

### 5.2. EEEV/VEEV/WEEV

Serological evidence exists supporting exposure of bats to encephalitic alphaviruses in the field, and experimental data demonstrate the susceptibility of bats to infection with alphaviruses including VEEV. Four Mexican bat species were examined for molecular evidence of infection with Venezuelan equine encephalitis virus (VEEV), Western equine encephalitis virus (WEEV), and Eastern equine encephalitis virus (EEEV). No individual bats were positive for WEEV or EEEV, but 3% (5/150) representing all four species were positive for VEEV [89]. Field-caught Jamaican fruit bats (*Artibeus jamaicensis*) and great fruit-eating bats (*Artibeus literatus*) were negative by PRNT for EEEV and WEEV antibodies, but 2.6% (1/38) had neutralizing antibodies to VEEV [66]. Similarly, the serum of 384 bats representing 14 species was subjected to ELISA, and 2.9% (11/384) contained VEEV-specific antibodies. ELISA and HI assays for EEEV and WEEV antibodies, respectively, were all negative [88]. Four species of wild-caught bats from the northeastern United States were tested for neutralizing antibody against EEEV and WEEV. Samples were negative for antibodies against WEEV, but 1.3% of the 128 bats tested did possess EEEV-neutralizing antibody [47]. Bats of the genera *Myotis* and *Eptesicus* were experimentally infected with EEEV, and developed viremia but failed to develop neutralizing antibodies. Infection of big brown bats by bite of *Culiseta melanura* and *Aedes aegypti* mosquitoes was successful. More non-hibernating than hibernating bats were seropositive for EEEV [146].

### 5.3. Other Emerging Alphaviruses

In a recent serosurveillance study, 2/432 bats were seropositive by plaque reduction neutralization assay to Babanki virus (BBKV) and 9/626 Egyptian rousette bats had non-specific alphavirus antibodies (Table 4) [29]. Multiple isolates of BBKV were obtained from *Cx. perfuscus* mosquitoes collected from multiple locations in Uganda during this same sampling period as when bats were sampled [147]. Mosquito blood meals from bats comprised 7.5% of the total blood meals identified from the species *Cx. perfuscus* [148]. It is unclear whether bats contribute to the transmission cycle of BBKV or are merely incidentally exposed through mosquito bites Ten *Pteropus poliocephalus* bats were experimentally infected with Ross River virus, and five developed low (log_10_ 2.2 TCID_50_/100 μL) detectable and short-lived (2 days) viremia. Still, 2% of the colonized mosquitoes (*Aedes vigilax*) that fed on the bats between days 1–4 post-infection became infected [148]. Kading et al. (2014) modeled that for viremias <log_10_ 2.0/mL, the probability of a mosquito becoming infected was around 0.1 or less given the low circulating titer and the volumetric constraints of a small mosquito blood meal; therefore, at least 10 mosquitoes would need to feed on an animal with a low viremia in order for one mosquito to ingest virus [10]. In the case of RRV, published data demonstrate that infection of mosquitoes fed on RRV-viremic bats is still possible despite a viremia and low titer [149]. Therefore, if bat and mosquito populations are in high numbers, 50% of bats develop a detectable viremia, and 2% of mosquitoes become infected, mosquito-borne transmission could take place even though experimentally-determined efficiencies are low [149]. Antibodies against Mayaro virus were detected by HI in 37 Trinidadian bat species tested [25].

## 6. Relationships between Bats and Arthropods

A number of hematophagous arthropods feed on bats, including bat flies (genera *Nycteribiidae* and *Streblidae*), bat bugs and bed bugs (family *Cimicidae*), and ticks (families *Argasidae* and *Ixodidae*) [18,20,21,167,168,169,170,171,172]. Viruses of medical and veterinary significance have also been isolated from these arthropods [21,173,174,175]. However, the contribution that these ectoparasites play in the circulation of medically important viruses among bats and other hosts is unclear and necessitates further investigation.

### 6.1. Mosquitoes

Kading and Schountz (2016) reviewed instances in the literature where mosquito blood meals have been identified as originating from bats [95]. Information on primary mosquito vectors feeding on bats is very limited. Tiawsirisup et al. 2012 collected mosquitoes from five genera inside a bat cave in Thailand to investigate sylvatic circulation of JBEV. While these collections included arbovirus vectors *Cx. quinquefasciatus* and *Cx. tritaenhiorhynchus*, the only blood-fed mosquitoes collected from the cave were *Cx. quinquefasciatus,* at least 20 of which had fed on Leschnault’s rousette (*Rousettus leschenaulti*) bats [176]. *Culiseta morsitans* Theobald mosquitoes (vector of Eastern equine encephalitis virus) were found to have fed on Eastern pipistrelle bats (*Pipistrellus subflavus*), but these blood meals comprised only 1% of the total blood meals identified from this mosquito species [177]. No information was found on any blood meals from bats being detected in *Aedes (Stegomyia)* species, but it is unclear how much investigation has been done in this area. Sixteen of 20 field-collected *Ae. funereus* mosquitoes (vector of RRV) had fed on *Pteropus alecto* bats [149]. In Africa, mosquito species in which bat blood meals have been identified and are known to be associated with a number of medically-important arboviruses include: *Coquillettidia (Cq.) fuscopennata* (Theobald) (YFV, Sindbis, chikungunya viruses), *Culex (Cx.) perfuscus* Edwards (WNV, Oropouche, Sindbis, Wesselsbron, Usutu, Babanki viruses), *Cx. (Cx.) neavei* Theobald (WNV, Babanki, Spondweni, Sindbis, Koutango viruses), and *Cx. (Cx.) decens group* (WNV, chikungunya, Babanki viruses [148,178]. While mosquitoes in the subgenus *Cx. (Cx.)* are recognized as primary vectors of WNV, Sindbis, Babanki, and Usutu viruses, only for Babanki virus has additional field data been collected so far that support a potential role for bats in virus circulation (discussed above) [29].

### 6.2. Bat Flies

Bat flies (Order: Diptera; Families: *Nycteribiidae*, *Streblidae*) are highly host-specific obligate hematophagus ectoparasites of bats [179,180]. A novel fusogenic orthoreovirus, tentatively named Mahlapitsi virus (MAHLV), was discovered in bat flies (*Eucampsipoda africana*) associated with Egyptian rousette bats [181]. A novel orthobunyavirus, tentatively named Wolkberg virus (WBV), was isolated from the same species of bat flies in a similar geographic region [182]. Dengue virus RNA was detected by RT-PCR in bat flies (*Strebla wiedemanni, Trichobius parasiticus*) associated with common vampire bats (*Desmodus rotundus*) [100]. *Bartonella* spp. bacteria have also been found infecting both fruit bats and the bat flies parasitizing them in Madagascar, providing a documented example of a pathogen-vector-bat association involving bat flies [183]. More research in this area is needed to elucidate the role of bat ectoparasites in spreading pathogens among individual bats in a roost.

### 6.3. Bat Bugs and other Arthropods in Family Cimicidae

In addition to parasitizing humans by infesting their dwellings, bed bugs and other arthropods in family *Cimicidae* are found in close association with bat populations [170]. Some cimicids are known to play a role in alphavirus transmission. Cliff swallow bugs (*Oeciacus vicarious*) transmit Fort Morgan virus and Buggy Creek virus [173,174,184,185] among passerine birds. Tonate virus, which is closely related to VEEV, and also causes fatal encephalitis in man, has also been isolated from swallow bugs in the 1970’s [186]. As previously described, bat bugs (*Cimex insuetus* and *Strcticimex parvus*) host the orthobunyavirus KKV which likely causes disease in humans [21,22], but this also remains a largely understudied research area.

### 6.4. Acari (Ticks and Mites)

Ticks and mites are known to parasitize bats and they both fill similar ecological niches. Many studies have identified viruses in bats that cluster phylogenetically with other arboviruses transmitted by ticks. Other isolations are indicative of bats playing a role in viruses isolated from either hard-bodied or soft-bodied ticks. For example, Estero Real Virus was first isolated from ticks (*Ornithodoros tadaridae*) obtained from a palm tree colonized by Cuban bats [187]. Due to the close ecological association between Egyptian rousette bats (*R. aegyptiacus*) and soft-bodied ticks in caves, the argasid tick *Ornithodoros faini* was screened for Marburgvirus RNA but all pools were found to be negative [19]. To date, evidence does not support the role of arthropods in the transmission of filoviruses in nature. Bats are also known to host mite species in families *Macronyssidae, Dermanyssidae*, and *Spinturnicidae*, though the significance of these arthropod’s role in virus transmission between bats and other vertebrates is largely unknown [188,189,190]. Species in *Macronyssidae* and *Dermanyssidae* have also been demonstrated to efficiently transmit arboviruses (further reviewed in [191]).

### 6.5. Other Bat-Arthropod Associations

A recent study explored viruses of potential arthropod-origin in the fruit bat *Hypsignathus monstrosus* and identified five viruses: one dicistrovirus (family *Dicistroviridae*, order *Picornavirales*), one nodavirus (family *Nodaviridae*), and two tombus-like viruses. They also detected a related tombus-like virus isolated from fig wasps and primitive crane flies co-habitating with bats, suggesting the ability of arthropods to host “bat-associated” viruses [192]. Tombus viruses typically infect plants, but tombus-like viruses infect a wide range of hosts, including arthropods and marine invertebrates [193,194,195]. Ingestion of arthropods by fruit bats has been documented [196,197], and could be a potential mechanism explaining the association between viromes of arthropods and frugivorous bat species.

## 7. Additional Considerations

With regard to experimental infections, one must take into account the method of inoculation (e.g., intracerebral, subcutaneous, intraperitoneal, intramuscular), the inoculating titer, as well as whether or not vector bites were associated with the infection. In analyzing serologic results, one must take into account whether the assay is detecting neutralizing or non-neutralizing antibodies, and consider the dynamics of antibody decay which may impact detection sensitivity [198]. Arthropod saliva has been shown to potentiate arbovirus infection, affecting not only the magnitude of peak viremia but also the viral loads in certain organs [199]. The inoculating viral strain used is also important, as it should align geographically with where the species under study resides to truly characterize their potential as a reservoir species. Komar et al. (2003) presented detailed methods for estimating the reservoir competence of vertebrate species for mosquito-borne viruses [200], including calculating a reservoir competence index based on the susceptibility to infection, mean daily infectiousness, and the duration of infectiousness. This reservoir competence index is interpreted as the relative number of infectious vectors that derive from a particular vertebrate species [200], and would be easily transferrable to evaluating the reservoir competence of bats. Further, bats are very long-lived (up to 25 years) compared to other small mammals, potentially increasing the chances of exposure and subsequent transmission to other vertebrates or invertebrate vectors [1]. Cell culture also offers a viable starting point for initial assessments of viral replication in a particular host species, particularly when in vivo studies are not feasible. To date, many cell lines derived from the organs of bats have been established [201].

Importantly, the intent of this review is not to vilify bats, but to analyze the existing literature and its support for bats as arboviral reservoirs, in addition to identifying areas for future study. Bats provide vital ecosystem services, such as arthropod suppression, pollination, and seed dispersal [202]. Past depopulation efforts in response to viral outbreaks resulted in increased viral spillover, and are not a viable means of disease control and have even resulted in higher virus infection rates in the bat species when colonies repopulated from neighboring roosts [203]. Further work should encompass directed field surveillance complemented by both in vitro and in vivo approaches, with field surveillance efforts focused on optimization of non-lethal and non-invasive sampling [204,205,206,207]. When possible, longitudinal sampling of identifiable animals through use of marking or tagging allows for monitoring of seroconversion and viremia persistence, providing a chance to better characterize transmission periodicity and seasonal changes in viremia profiles.

## 8. Conclusions

To truly elucidate the role of bats as reservoirs for arboviruses, field surveillance studies documenting natural infection and transmission dynamics among vector and vertebrate species must be supplemented with experimental infections to characterize viremia profiles and infectiousness to vectors, virus-induced pathology, and immune kinetics following infection. With bats, these tasks are not trivial, and carry significant challenges in both the field and the lab. These challenges are evidenced by the few arboviruses for which there are substantial field and laboratory data involving the infection of bats. While many studies have presented serologic data indicative of past exposure of free-ranging bats to arboviruses of medical and veterinary importance, these studies should be followed up with laboratory assessments of reservoir host competence to shed light on the true epidemiological significance of the field data. Further, the detection of viral nucleic acid in free-ranging bats does not necessarily implicate the species as an arboviral reservoir. Rather, recovery and isolation of live virus at biologically relevant titers, and demonstrating the persistence of the pathogen in nature among connected populations of a potential reservoir species is more definitive. Unfortunately, few established bat colonies exist for use in vivo viral pathogenesis studies, limiting the achievability of these studies. 

To fulfill the vertebrate reservoir host paradigm, in vivo infections must support findings in the field. The isolation of Marburg virus from Egyptian rousette bats in Uganda in addition to experimental infections demonstrating viremia and shedding in the absence of overt pathology support the role of this bat species as the reservoir for Marburg virus [6,7,208]. For arboviruses, the combination of field work and in vivo pathogenesis studies are lacking. Still, several examples have emerged from this review that point to bats as potentially competent amplifying hosts for arboviruses. Kaeng Khoi virus was isolated from both bats and cimicid bugs in Thailand, and was also shown to be the causative agent behind sick mine workers [21]. Some bat species did develop a viremia at a level that would be infectious to mosquitoes when inoculated experimentally with CHIKV, and bats have been found exposed to CHIKV during field investigations [66,143]. Experimental evidence supporting transmission of RRV from *Pteropus poliocephalus* to recipient mosquitoes in addition to identification of RRV-seropositive bats trapped in Australia and Indonesia highlights the need for further investigation into the role of bats in the ecology of this disease [75,149,157]. A mosquito-bat-mosquito transmission cycle was established in the lab for JBEV [67]. Serological evidence from the field documenting bat exposure to JBEV, the sustained viremia in bats during hibernation, and demonstration of mosquito transmission from and to bats in the laboratory collectively demonstrate the capability of bats to function as reservoir hosts for this virus. Circumstantial evidence from field-sampled mosquitoes and bats supports the cycling of Babanki virus among bats and mosquitoes in Uganda [29], but experimental data are still lacking. For RVFV, bats evaluated in the lab have supported virus replication, and multiple populations of bats in the field have been found with neutralizing antibodies or natural infection with RVFV [1,28,29]. Additional studies are warranted on these and other viruses for which additional field or experimental data are needed to support the role of bats as a possible reservoir.

## Figures and Tables

**Table 1:** Table describing species with published results describing virus isolation, molecular evidence, or seroconversion to species in family *Bunyavirales*.

Family	Virus	Virus Isolation/ Molecular Evidence	Serologic Evidence	Ref(s)
*Nairoviridae*, genus *Orthonairovirus*	Ahun virus	*Myotis mystacinus, Pipistrellus pipstrellus*		[34]
	Crimean-Congo Hemorrhagic Fever Virus (CCHF)		*Rousettus aegyptiacus, Coleura afra, Hipposideros cf. caffer, Miniopterus inflatus, Hipposideros gigas, Eidolon helvum, Epomops franqueti, Hypsignathus monstrosus, Micropteropus pusillus, Myonycteris torquata, Myotis dasycneme, Myotis daubentonii, Myotis blythii, Nyctalus noctula* Unidentified species (France)	[15,16,17]
	Gossas (GOSV)	*Tadarida* sp.		[35]
	Issyk-Kul (IKV)	*Nyctalus noctula*, *Myotis blythii*, *Vespertilio serotinus*; Argasid ticks collected from *Vespertilio pipstrellus, V. serotinus, Nyactulus noctula,* and *Myotis blythii*		[35,36]
	Kasokero (KKOV)	*Rousettus aegyptiacus*		[35,37,38]
	Keterah (KTRV)	Tick larvae (*Argus pusillus*) collected from *Scotophilus temmincki*		[39]
	Leopards Hill (LPHV)	*Hipposideros gigas*		[35,40]
	Uzun Agach (UAV)	*Myotis blythii*		[41]
	Yogue (YOGV)	*Rousettus aegyptiacus*		[32,35,38]
*Peribunyaviridae,* genus *Orthobunyavirus*	Bunyamwera virus (BUNV)	*Myotis lucifugus, Eidolon helvum, Rousettus aegyptiacus, Mops condylurus*	*Eidolon helvum, Rousettus aegyptiacus, Mops condylurus*	[42,43,44,45,46]
	Bimiti virus (BIMV)		*Anoura geoffroyi, Carollia perspicillata, Phyllostomus hastatus, Pteronotus parnellii, Natalus tumidirostrus*	[25]
	California encephalitis virus (CEV)		*Myotis keenii*	[47]
	Catú virus (CATUV)	*Molossus obscurus*	*Anoura geoffroyi, Carollia perspicillata, Phyllostomus hastatus*	[25]
	Guama virus (GMAV)	Unidentified bat	*Anoura geoffroyi, Phyllostomus hastatus, Artibeus literatus*	[25,32]
	Kaeng Khoi Virus (KKV)	*Chaerephon plicata*	*Taphazous theobaldi, Chaerephon plicata*	[21,22,48,49]
	Manzanilla virus (MANV)		*Molossus ater*	[25]
	Nepuyo virus (NEPV)	*Artibeus jamaicensis*, *Artibeus literatus*	*Artibeus jamaicensis, Phyllostomus hastatus*	[24,25]
	Oriboca virus (ORIV)		*Artibeus literatus*	[25]
	Restan virus (RESV)		*Artibeus literatus, Artibeus jamaicensis, Carollia perspicillata*	[25]
*Phenuiviridae,* genus *Phlebovirus*	Malsoor virus	*Rousettus leschenaultia*		[50]
	Rift Valley fever virus (RVFV)	*Miniopterus schreibersii, Eptesicus capensis, Micropteropus pusillus, Hipposideros abae, Hipposideros caffer, Epomops franqueti, Glauconycteris argentata*	*Rousettus aegyptiacus, Epomophorus labiatus*	[1,27,29,51,52,53]
	Toscana virus (TOSV)	*Pipistrellus kuhli*		[54,55]
Unclassified	Bangui virus (BGIV)	Unidentified bat		[31]
	Mojuí dos Campos virus (MDCV)	Unidentified bat		[32,33]

**Table 2:** Table describing species with published results describing virus isolation, molecular evidence, or seroconversion to species in genus *Flavivirus* (family *Flaviviridae*).

Virus	Virus Isolation/ Molecular Evidence	Serologic Evidence	Ref(s)
Banzi virus (BANV)		*Eidolon helvum, Epomophorus anuras, Miniopterus schreibersii, Tadarida pumila, Mops condylurus*	[44]
Bussuquara virus (BSQV)		*Artibeus jamaicensis*	[66]
Central European encephalitis virus	Unidentified bat		[1]
Dengue virus (DENV)	*Desmodus rotundus, Artibeus jamaicensis, Carollia brevicauda, Myotis nigricans, Glossophaga soricina, Artibeus literatus, Artibeus planirostris, Carollia perspicillata, Myotis lucifugus, Artibeus intermedius, Molossus sinaloae, Molossus pretiosus, Rhogeessa bickhami, Molossus rufus, Eumops glaucinus*	*Myotis nigricans, Pteronotus parnellii, Natalus stramineus, Artibejus jamaicensis, Artibeus* spp., *Uroderma* spp., *Molossus* spp., *Chaerephon pumilus, Mops condylurus, Anoura geoffroyi, Artibeus cinereus, Artibeus literatus, Carollia perspicillata, Molossus ater, Molossus molossus, Phyllostomus hastatus, Pteronotus davyi, Pteronotus parnellii, Sturnira* spp., *Pteropus gouldii, Pteropus giganteus, Glossophaga soricina, Artibeus intermedius, Molossus sinaloae, Rhogeessa io, Molossus pretiosus, Balantiopteryx plicata, Molossus rufus, Rhogeessa bickhami, Epomophorus labiatus*	[25,29,57,59,60,61,62,63,65,78,97,100,101,102]
Ilheus virus (ILHV)		*Anoura geoffroyi, Phyllostomus hastatus, Pteronotus davyi, Artibeus jamaicensis, Artibeus literatus, Desmodus rotundus, Molossus ater*	[25]
Japanese encephalitis virus (JBEV)	*Murina aurata*, *Rousettus leschenaultia, Eptesicus fuscus, Myotis lucifugus, Pipistrellus subflavus, Pipistrellus abramus, Tadarida brasiliensis, Hipposideros armiger terasensis, Miniopterus fuliginosus, Rhinolophus cornutus, Miniopterus schreibersii, Rhinolophus cornutus, Pteropus alecto, Cynopterus sphinx*	*Rousettus leschenaultia*, *Taphozous melanopogon*, *Miniopterus fuliginosus, Myotis macrodactylus*, *Miniopterus shreibersii, Eptesicus fuscus, Pteropus alecto, Pteropus goldii, Pteropus scapulatus,* Genera *Hipposideros* and *Miniopterus, Pteropus giganteus, Murina leucogaster, Megaderma lyra, Cynopterus sphynx, Myotis mystacinus, Pipistrellus abramus, Plecotus auritus, Rhinolophus ferrum-equinum, Vespertilio superans, Hipposideros armiger, Hipposideros pomona, Hipposideros speoris, Hipposideros bicolor, Hipposideros cineraceus, Rhinolophus cornutus, Rhinolophus rouxi, Rousettus leschenaultia, Miniopterus schreibersii, Pipistrellus abramus, Rhinolophus macrotus,* undetermined species	[32,67,69,70,71,72,73,74,75,76,77,78,79,80,81,82,83,87,103,104,105,106]
Jugra virus (JUGV)	*Cynopterus brachyotis*		[32]
Kyasanur forest disease virus (KFDV)	*Rhinolophus rouxi*, *Cynopterus sphinx*	*Rousettus leschenaultii, Cynopterus sphinx, Pteropus giganteus, Rhinolophus rouxi*	[107,108,109]
Murray Valley encephalitis virus (MVEV)		*Eptesicus pumilus, Pteropus gouldi, Pteropus scapulatus, Pteropus* spp.	[78,102,110,111]
Ntaya virus (NTAV)		*Eidolon helvum, Rousettus* sp.	[43,46]
St. Louis encephalitis virus (SLEV)	*Eptesicus fuscus, Myotis lucifugus, Tadarida brasiliensis*	*Artibeus intermedius, Artibeus jamaicensis, Artibeus literatus, Artibeus phaeotis, Glossophaga soricina*, *Molossus major*, *Phyllostomus hastatus, Sturnira lilium, Eptesicus fuscus, Myotis lucifugus, Molossus ater, Anoura geoffroyi, Carollia perspicillata, Molossus molossus, Natalus tumidirostris, Pteronotus davyi, Pteronotus parnellii, Tadarida brasiliensis*, *Sturnira* spp.	[25,69,69,85,87,88,97,103,112,113,114,115,116]
Tick-borne encephalitis virus (TBEV)	*Myotis myotis, Barbastella barbastellus, Plecotus auritus*	*Barbastella barbastellus, Myotis myotis, Plecotus auritus, Rhinolophus hipposideros*	[117,118,119]
Israel turkey meningoencephalitis (ITV)		*Rousettus aegyptiacus*	[120]
Uganda S virus (UGSV)		Unspecified	[121]
Usutu virus (USUV)	*Rousettus aegyptiacus*	*Eidolon helvum*, *Rousettus* sp.	[43,46]
West Nile virus (WNV)	*Rousettus leschenaultia, Rousettus aegyptiacus*	*Eptesicus fuscus, Myotis lucifugas, Myotis septentrionalis, Eidolon helvum, Epomophorous minor, Pteropus scapulatus, Mops condylurus, Glossophaga soricina, Tadarida pumila, Rousettus* sp., *Rousettus aegyptiacus*, Undetermined species, *Artibeus jamaicensis, Artibeus literatus*	[29,43,44,46,78,86,90,97,120,121,122]
Yellow fever virus (YFV)	*Eidolon helvum, Rousettus aegyptiacus, Mops condylurus, Epomophorus* sp., *Eptesicus fuscus, Myotis lucifugus*	*Eidolon helvum, Rousettus aegyptiacus, Phyllostomus hastatus, Artibeus cinereus, Artibeus jamaicensus, Artibeus literatus, Carollia perspicillata, Glossophaga soricina, Molossus ater, Molossus molossus, Phyllostomus hastatus, Pteronotus davyi, Pteronotus parnellii, Vampyrops helleri, Mops condylurus, Tadarida pumila, Epomophorus* sp., *Rousettus* sp.	[25,29,43,44,45,46,68,121,123,124,125,126]
Zika virus (ZIKAV)	*Eidolon helvum, Rousettus aegyptiacus, Rousettus angolensis*	Undetermined species, *Rousettus aegyptiacus*, *Mops condylurus, Tadarida pumila, Eidolon helvum, Rousettus* sp., Unspecified	[43,44,46,75,121]

**Table 3:** Table describing species with published results describing virus isolation, molecular evidence, or seroconversion to species in Family *Reoviridae*.

Genus	Virus	Virus Isolation/ Molecular Evidence	Serologic Evidence	Ref(s)
*Coltivirus*	Taï Forest reovirus (TFRV)	*Chaerephon aloysiisabaudiae*		[127]
*Orbivirus*	Bukakata (BUKV)	*Rousettus aegyptiacus*		[129]
	Elsey virus (PHSV)		*Pteropus* spp.	[131]
	Fomede (FOMV)	*Nycteris nana, Nycteris gambiensis*		[52,132,133,134]
	Heramatsu virus	*Myotis macrodactylus*		[135,136]
	Ife (IFEV)	*Eidolon helvum*		[137,138]
	Japanaut (JAPV)	*Syconycteris crassa*		[139,140]
	Matucare virus (MATV)		Genera *Myotis* and *Noctilio*	[141]

**Table 4:** Table describing species with published results describing virus isolation, molecular evidence, or seroconversion to species in genus Alphavirus (family Togaviridae).

Virus	Virus Isolation/ Molecular Evidence	Serologic Evidence	References
Babanki virus (BBKV)		*Epomophorus labiatus, Rousettus aegyptiacus*	[29]
Chikungunya (CHIKV)	*Eptesicus fuscus, Rousettus leschenaultia, Pteropus giganteus, Scotophilus* sp., *Rousettus aegyptiacus, Chaerephon pumilus*	*Artibeus literatus*, *Artibeus jamaicensis, Pteropus giganteus, Megaderma lyra Hipposideros cafer* undetermined species	[1,44,60,66,75,143,150,151,152,153]
Eastern equine encephalitis virus (EEEV)	*Eptesicus fuscus, Myotis lucifugus, Myotis keenii*	*Artibeus intermedius, Artibeus jamaicensis, Artibeus literatus, Glossophaga soricina, Rhynchonycteris naso, Sturnira lilium, Carollia perspicillata, Phyllostomus hastatus, Vampyrops helleri, Eptesicus fuscus, Myotis lucifugus, Myotis keenii*	[25,44,47,67,112,146,154,155]
Mucambo virus (MUCV)		*Molossus ater, Phyllostomus hastatus, Carollia perspicillata*	[25,156]
O’Nyong Nyong virus (ONNV)		*Rousettus aegyptiacus, Chaerephon pumila*	Kading, pers comm
Ross River virus (RRV)	*Pteropus poliocephalus*	*Pteropus poliocephalus*, *Pteropus scapulatus,* undetermined species	[75,149,157]
Sindbis virus (SINV)	*Rhinolophidae* sp., *Hipposiderae* sp., *Myotis lucifugus, Eidolon helvum*		[1,43,158,159]
Semliki Forest Virus (SFV)	*Myotis lucifugus, Eidolon helvum, Rousettus aegyptiacus, Mops condylurus*	*Eidolon helvum, Rousettus aegyptiacus, Mops condylurus*	[42,43,44,158]
Venezuelan equine encephalitis virus (VEEV)	*Carollia perspicillata, Eptesicus fuscus, Artibeus planirostris, Sturnira lilium, Artibeus turpis, Desmodus rotundus, Artibeus literatus, Artibeus literatus, Carollia sowelli, Sturnira parvidens, Glossophaga soricina, Uroderma bilobatum, Artibeus phaeotis, Pipistrellus subflavus, Plecotus townsendii*	*Artibeus* sp., *Carollia brevicauda, Carollia subrufa, Carollia perspicillata*, *Desmodus rotundus*, *Glossophaga soricina*, *Noctilio leporinus, Sturnira lilium, Sturnira ludovici, Artibeus jamaicensis, Artibeus literatus, Phyllostomus discolor*	[1,32,66,88,89,112,160,161,162,163,164,165]
Western equine encephalitis virus (WEEV)		*Artibeus jamaicensis*	[166]
